# Role of SOD3 in silica-related lung fibrosis and pulmonary vascular remodeling

**DOI:** 10.1186/s12931-018-0933-6

**Published:** 2018-11-20

**Authors:** Igor N. Zelko, Jianxin Zhu, Jesse Roman

**Affiliations:** 1 Department of Medicine, Division of Pulmonary, Critical Care, and Sleep Medicine, University of Louisville, 505 S. Hancock Street, CTR Bldg., room 524, Louisville, KY 40202 USA; 2 Department of Biochemistry and Molecular Genetics, University of Louisville, 505 S. Hancock Street, CTR Bldg., room 524, Louisville, KY 40202 USA; 3 Department of Pharmacology and Toxicology, University of Louisville Health Sciences Center, Louisville, KY 40202 USA; 4 Robley Rex VA Medical Center, Louisville, KY 40202 USA

**Keywords:** Oxidative stress, Transcription, Expression, Crystalline silica, Pulmonary hypertension, Lung fibrosis, Silicosis, Pneumoconiosis

## Abstract

**Background:**

Work-place exposure to silica dust may lead to progressive lung inflammation culminating in the development of silicosis, an irreversible condition that can be complicated by onset of pulmonary hypertension (PH). The molecular mechanisms leading to the development of PH and lung fibrosis in response to silica are not well understood. Oxidant/antioxidant imbalance in the lung may promote fibroproliferation and vascular smooth muscle proliferation, ultimately leading to the development of PH. Herein, we analyze the development of PH and lung fibrosis in mice deficient in extracellular superoxide dismutase (SOD3), an enzyme with anti-oxidant activity.

**Methods:**

PH and silicosis were induced in wild-type and Sod3^−/−^ mice through intratracheal injection of crystalline silica at dose 0.4 g/kg. Pulmonary hypertension and lung fibrosis were characterized by changes in right ventricular systolic pressure (RVSP) and collagen deposition 28 days following silica injections. Vascular remodeling was analyzed using immunohistochemistry and morphometric analysis. The expression of genes were analyzed using qRT-PCR and Western blot.

**Results:**

C57BL6 mice exposed to silica showed attenuated expression of Sod3 in the lung suggesting a protective role for Sod3. Consistent with this, Sod3^−/−^ mice developed more severe fibrotic inflammatory nodules with increased collagen deposition. Furthermore, the expression of genes involved in tissue remodeling (Timp1), fibrotic lesion formation (Fsp1) and inflammatory response (Mcp1) were significantly elevated in Sod3^−/−^ mice compared to Sod3^+/+^ mice treated with silica. Infiltration of neutrophils and activated macrophages into affected lung was significantly higher in Sod3 deficient mice. In addition, silica produced more profound effects on elevation of RVSP in Sod3^−/−^ compared to wild-type littermate. Increase in RVSP was concomitant with hypertrophy of pulmonary arteries located in silicotic nodules of both mouse strains, however, vascular remodeling in unaffected areas of lung was detected only in Sod3^−/−^ mice.

**Conclusions:**

Our data suggest that Sod3 and extracellular oxidative stress may play an important role in the development of pneumoconiosis and pulmonary vascular remodeling following exposure to environmental and occupational silica.

## Introduction

The occupational exposure to crystalline silica during coal mining, sand blasting, volcanic eruption and wind erosion remains a significant hazard worldwide. Crystalline silica inhalation results in the development of silicosis and pulmonary fibrosis, which eventually results in reduced lung function and other health related issues [1, 2]. In addition to silicosis, the chronic inhalation of crystalline silica promotes the development of chronic obstructive pulmonary diseases (COPD) and lung cancer [3, 4]. Several animal models were developed to investigate the effect of silica dust on inflammatory responses in the lung and to elucidate the molecular mechanisms responsible for the pro-fibrotic effects of silica crystals [5, 6]. Crystalline silica trapped in lung alveoli induces inflammatory responses that eventually lead to fibronodular parenchymal lung disease characterized by excessive deposition of extracellular matrix proteins and accumulation of fibroblasts, epithelioid inflammatory cells, and cellular fragments [7, 8]. Epidemiological data indicate that patients with silicosis and other parenchymal lung diseases are frequently diagnosed with elevated pulmonary pressure [9, 10]. Moreover, increased pulmonary pressure above 25 mmHg is a strong indicator of worst outcome in patients with silicosis and other underlying interstitial lung diseases [11, 12].

The pathogenesis of interstitial lung diseases and pulmonary hypertension is incompletely understood, although recent literature points to the important role of an oxidant/antioxidant imbalance in the development of these serious disorders. Since crystalline silica is a strong promoter of inflammatory responses in pulmonary cells, mostly through the induction of a burst of reactive oxygen species in affected areas of the lung, we decided to explore the role of an antioxidant enzyme in the development of lung fibrosis and pulmonary vascular remodeling leading to pulmonary hypertension in mice treated with silica [13]. One of the most abundant antioxidant enzymes in the pulmonary vasculature is extracellular superoxide dismutase (SOD3). In the human vessel wall, SOD3 is expressed at the highest level, comprising more than half of the total vascular SOD, with activity levels ~ 10-fold higher than in other tissues [14, 15]. Vascular SOD3 interacts with heparin and other extracellular matrix proteins on the surface of endothelial and smooth muscle cells and is involved in the regulation of vascular tone under physiological conditions and during oxidative stress. It has been shown that SOD3 preserves endothelium-dependent relaxation and reduces angiotensin-induced blood pressure [16, 17]. Moreover, genetic manipulation of SOD3 expression affects the development of pulmonary hypertension in mice exposed to bleomycin or chronic hypoxia [18–20]. SOD3 activities and expression are also dysregulated during development and progression of atherosclerosis [21] and hypertension [22, 23].

In this study, we investigated the protective role of SOD3 in silica-induced lung fibrosis and in pulmonary vascular remodeling and development of pulmonary hypertension. We found that silica exposed Sod3^−/−^ mice developed more lung fibrosis, increased thickening of vascular walls in remodeled pulmonary vessels, and higher right ventricular pressure (RVSP) when compared to wild-type littermate.

## Material and methods

### Experimental animals and animal care

C57BL/6 J mice were obtained from Jackson Laboratory (Bar Harbor, ME). The Institutional Animal Care and Use Committee of the University of Louisville approved the research protocol #15129, and the care and handling of the animals were in accordance with the National Institutes of Health guidelines.

### Animal model

SOD3 knockout mice (Sod3^−/−^) in the C57BL/6 J background were described previously [24]. Littermate wild-type C57BL/6 J were used as control. Adult male Sod3^+/+^ and Sod3^−/−^ mice (10 weeks of age) were separated into 3 experimental groups with 5 animals per group. Animals were anesthetized and placed in the supine position. Using sterile technique, the trachea was exposed via midline neck incision followed by instillation of silica or saline as described previously [25]. Crystalline silica was sterilized at 200 °C for 2 h to inactivate endotoxin contamination. Silica suspension in sterile 0.9% NaCl was prepared by vigorous vortexing immediately prior to intratracheal administration. Using a 27-gauge needle attached to a microliter syringe, 0.4 g/kg of crystalline silica suspension or the equivalent volume of saline was instilled into the trachea. The incision was then closed using surgical clips, and animals were allowed to recover. Twenty-eight days after intratracheal instillation of silica, RSVP parameters were measured and lung tissue were harvested for morphological, biochemical, and histochemical analyses. Tissue was immediately processed or quick-frozen in liquid nitrogen.

### Reagents

Primers and probes for real-time PCR were obtained from Integrated DNA Technologies (Coralville, IA) and ThermoFisher Scientific (Waltham, MA). All other chemicals and enzymes were from Sigma Chemical Co. (St. Louis, MO), or Invitrogen (Carlsbad, CA).

### Hemodynamic measurements

RVSP was determined with a 1F pressure transducer catheter (Millar Instruments). Briefly, the 1F pressure transducer was inserted through the right external jugular vein of anesthetized mice (100 mg ketamine/5 mg xylazine/kg of body weight, i.p.). Mice were placed on thermal plates to keep body temperature constant at 37 °C. Then, a pressure catheter was threaded into the right ventricle and RVSP was recorded using PowerLab 4/35 (ADInstruments) and analyzed using LabChart 8 software.

### Lung histology and Histomorphometric analysis

Lungs were flushed with PBS and inflated and fixed with 10% formalin overnight, then embedded in paraffin, sectioned at a thickness of 5 μm, and stained with Mason’s trichrome to visualize lung morphology and fibrosis. Images of lung sections were acquired using a high-resolution digital camera connected to a light microscope. Histomorphometric analysis of collagen deposition in silicotic nodules was performed using image analysis. At least six images per slide were captured at 400x magnification. The color deconvolution ImageJ software was used to evaluate the percentage of blue stained collagen in the image area. This software recognizes the image colors and decomposes them in three basic colors: blue (collagen), red, and purple. The morphometric analysis, corresponding to the blue color, was measured as the percentage of the total pixels in each image using the “threshold color” (ImageJ software), as described previously [26]. Collagen quantification was performed by an investigator blinded to treatment groups.

### Immunohistochemical staining of mouse lungs for smooth muscle, von Willebrand factor (vWF), and LY-6B

Longitudinal sections (5 μm) of left lung lobe were hydrated and antigen retrieval was first performed by incubating with 0.1% pronase for 5 min at 37 °C and then heating the slides in 10 mM sodium citrate (pH 6.0) plus 0.05% Tween 20 at 98 °C for 10 min. Sections were stained with anti-smooth muscle actin-alpha antibodies clone 1A4 (Sigma) at concentration 23 ng/μl, anti-vWF antibodies H-300 (Sigma) and anti-LY-6B antibodies (Bio-Rad) at concentration 10 ng/μl. After washing, the slides were incubated with secondary antibodies labeled with either AlexaFluor 488 or AlexaFluor 594. To determine the specificity of staining, lung sections were incubated with control, non-immune IgG. Slides were analyzed with fluorescent microscopy.

### Measurement of medial wall thickness

The hypertrophy of medial wall was measured in small muscularized arteries (< 200 μm) and expressed as a ratio of α-SMA stained area to perpendicular lumen radius. The measurements were performed using ImageJ (National Institutes of Health, Bethesda, MA). Pulmonary arteries were defined as vessels that accompanied airways (veins are interlobular). Five to ten pulmonary vessels were measured for each mouse. The analysis was performed by an investigator blinded to treatment groups.

### Quantitative RT-PCR

Total RNA was prepared from lung using RNAqueous-Micro Kit (Applied Biosystems, Foster City, CA). The synthesis of single stranded DNA from RNA was performed using SuperScript First-Strand Synthesis System for RT-PCR and random hexamers (Invitrogen, Carlsbad, CA), according to the protocol provided by manufacturer. To quantitate the abundance of gene-specific mRNAs, quantitative PCR was undertaken using the StepOnePlus Real-Time PCR Detection System (Applied Biosystems, Foster City, CA) and an SYBR® Green Master Mix. The PCR cycles were 95 °C for 3 min, then 40 cycles of 95 °C for 15 s, 60 °C for 1 min. PCR assays were run in triplicate, and gene expression was normalized to β-Actin mRNA levels. The mouse Monocyte Chemotactic Protein 1 (Mcp1) primers were forward (5′- GAA GGA ATG GGT CCA GAC AT -3′) and reverse (5’-ACG GGT CAA CTT CAC ATT CA-3′), mouse Fibroblast-specific Protein 1 (Fsp1) primers were forward (5′-AGG AGC TAC TGA CCA GGG AGC T-3′) and reverse (5’-TCA TTG TCC CTG TTG CTG TCC-3′), mouse Sod3 primers were forward (5’-GGC AAC TCA GAG GCT CTT C-3′) and reverse (5′-GTA GCA AGC CGT AGA ACA AGA-3′), mouse endothelin 1 (ET-1) primers were forward (5’-TCT GCA CTC CAT TCT CAG C-3′) and reverse (5’-CGT GAT CTT CTC TCT GCT GTT C-3′),), mouse insulin growth factor 1 (IGF-1) primers were forward (5′-GAG ACT GGA GAT GTA CTG TGC-3′) and reverse (5’-CTC CTT TGC AGC TTC GTT TTC-3′),), mouse platelet factor 4 (PF4) primers were forward (5’-ACC ATC TCC TCT GGG ATC CAT-3′) and reverse (5’-CCA TTC TTC AGG GTG GCT ATG AG-3′), mouse platelet/endothelial cell adhesion molecule 1 (Pecam1) primers were forward (5′-AGA GAC GGT CTT GTC GCA GT-3′) and reverse (5’-TAC TGG GCT TCG AGA GCA TT-3′). PCR assays were run in triplicate, and gene expression was normalized to β-Actin mRNA levels. Primers for β-Actin were forward (5′- ACA GCT TCT TTG CAG CTC CT-3′) and reverse (5’-CCA TCA CAC CCT GGT GCC TA-3′). Analysis of Collagen Type 1 Alpha 1 chain (Col1a1), Tissue Inhibitor of Metalloproteases 1 (Timp1), Connective Tissue Growth Factor (Ctgf), Matrix Metallopeptidase 2 (Mmp2) Cu,Zn-Superoxide Dismutase (Sod1), and Mn-Superoxide Dismutase (Sod2) were performed using custom gene expression assays with FAM labeled probe obtained from Applied Biosystems. The mRNA levels for these genes were normalized for β-Actin mRNA levels that were detected with custom gene expression assay with VIC labeled probe.

### Data analysis

All values are presented as means ± SEM of data from *n* independent experiments, as indicated in the figure legends. The statistical significance of differences was determined by t-test. A one-way analysis of variance (ANOVA) with Holm-Sidak post hoc test was used to compare differences between multiple treatment groups. *P*-value of < 0.05 indicated statistically significant differences.

## Results

### Silica exposure is associated with reduction in SOD3 expression

**Fig. 1:**
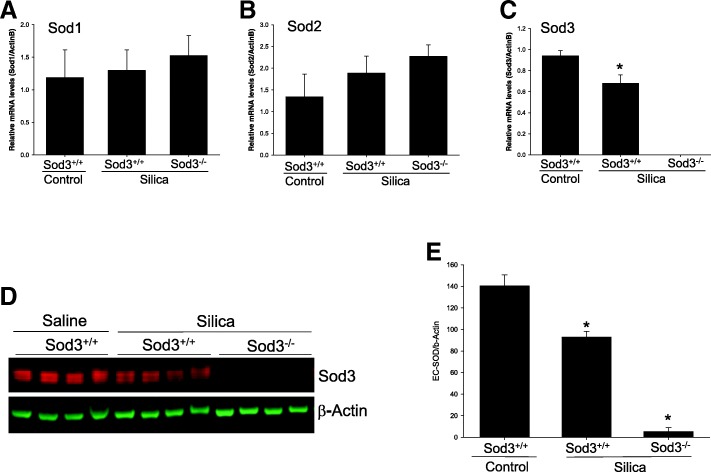
Attenuation of SOD3 gene expression in the lung of silica treated mice. **a-c** Relative mRNA levels for three SOD isoforms were determined using quantitative RT-PCR and normalized to β-actin expression. **d** Western blot of lung tissues isolated from control and silica treated mice. Beta-actin was used as a control. **e** Quantitative analysis of SOD3 protein normalized to β-actin expression. The results are shown as means ± SEM, *p < 0.05 when compared to control lung, One Way ANOVA with Holm-Sidak post test

In order to explore the role of oxidative stress and superoxide dismutases in the development of pulmonary hypertension and silicosis, we began by analyzing the expression of three isoforms of superoxide dismutases in the lungs of control and silica-treated wild-type mice. We found that while mRNA levels of SOD1 and SOD2 did not change after exposure to silica, the expression of SOD3 gene was significantly attenuated by 27.7% in the lungs of silica-treated mice (Fig. 1a-c). Similar reductions in SOD3 protein levels were detected (Fig. 1d, e) suggesting reduced antioxidant protection in the lung 28 days after exposure to inhaled silica.

### Silica exposure in the setting of SOD3 deficiency results in increased weight loss, enhanced lung lesions, and alterations in the expression of proinflammatory and tissue remodeling genes

**Fig. 2:**
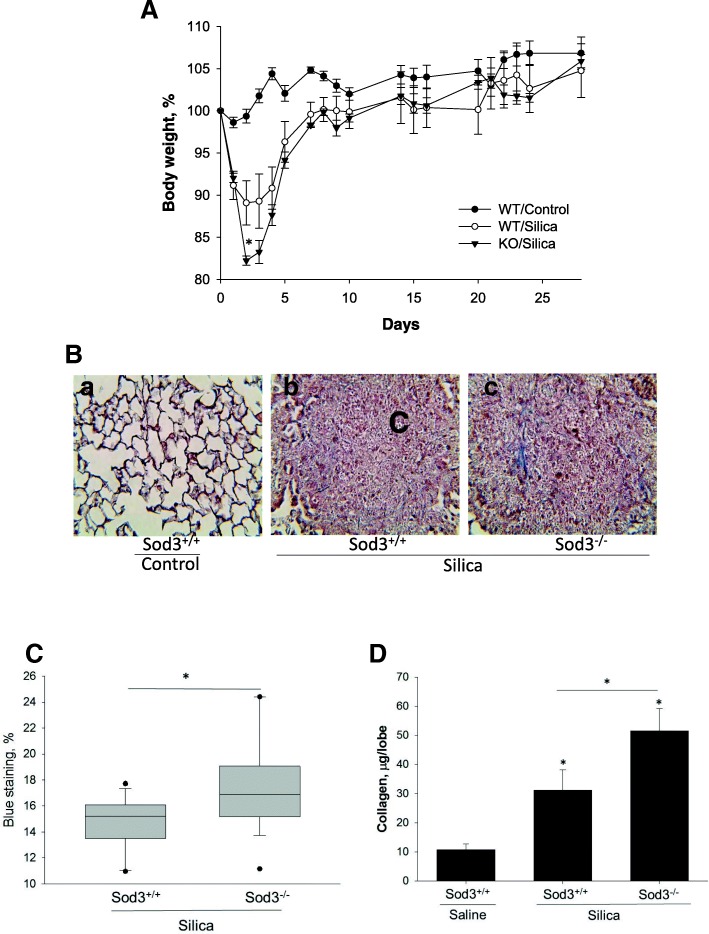
Weight loss and right ventricular systolic pressure (RVSP) measurements in mice treated with silica. Deficiency of SOD3 increased body weight loss in early stage of silica-induced lung fibrosis. **a** Effect of SOD3 gene knockout on body weight loss after intratracheal injection of crystalline silica. Body weight was measured for 28 days. *p < 0.05 for silica-challenged Sod3^−/−^ versus silica-challenged Sod3^+/+^ mice. **b** Histochemical analysis of silicotic nodules in the lung of Sod3^+/+^ and Sod3^−/−^ mice treated with crystalline silica. 40x magnification**. c** Analysis of collagen specific blue staining. *t*-test, * < 0.05. **d** Collagen accumulation in the lung. The results are shown as mean ± SEM, *p < 0.05 when compared to control lung, One Way ANOVA with Holm-Sidak post test

Considering the observations in wild-type mice, we set out to evaluate the effect of silica exposure in wild-type and Sod3^−/−^ mice. We found that the inhalation of crystalline silica induced an acute inflammatory response in the lung that led to the loss of body weight in affected animals. At day 2 following intratracheal silica administration (0.4 g/kg), Sod3^−/−^ mice lost significantly more body weight (− 10.9 ± 5.84% for Sod3^+/+^ and − 17.8 ± 1.22% for Sod3^−/−^, *p* < 0.05) compared to wild-type littermates (Fig. 2a). However, the differences in body weight between Sod3^+/+^ and Sod3^−/−^ mice became non-significant by day 5. At day 28, both strains of mice recovered their body weight and reached the average weight of wild-type untreated animals.

To analyze the effect of intratracheal instillation of silica on lung structure, lung tissues were fixed, stained with Mason’s trichrome and examined using light microscopy. Control mice showed no obvious abnormalities in lung structure, while mice challenged with silica developed noticeable fibrotic nodules with increased collagen deposition at day 28 (Fig. 2b). The quantitative analysis of fibrotic changes in the lung indicated that both Sod3^+/+^ and Sod3^−/−^ mice showed increased deposition of soluble collagen. The total amount of collagen in wild-type increased from 10.73 ± 1.94 μg/lobe in control mice to 31.19 ± 6.87 μg/lobe collagen in silica treated animals. Sod3^−/−^ mice showed significantly higher (51.54 ± 7.62 μg/lobe) collagen accumulation in the lung (Fig. 2d). Similar results were obtained when collagen specific blue staining was analyzed in fibrotic nodules of Sod3^+/+^ and Sod3^−/−^ mice (Fig. 2b, c). These data suggest that expression of SOD3 might protect the lung from silica-induced accumulation of collagen and the formation of fibrotic nodules.

**Fig. 3:**
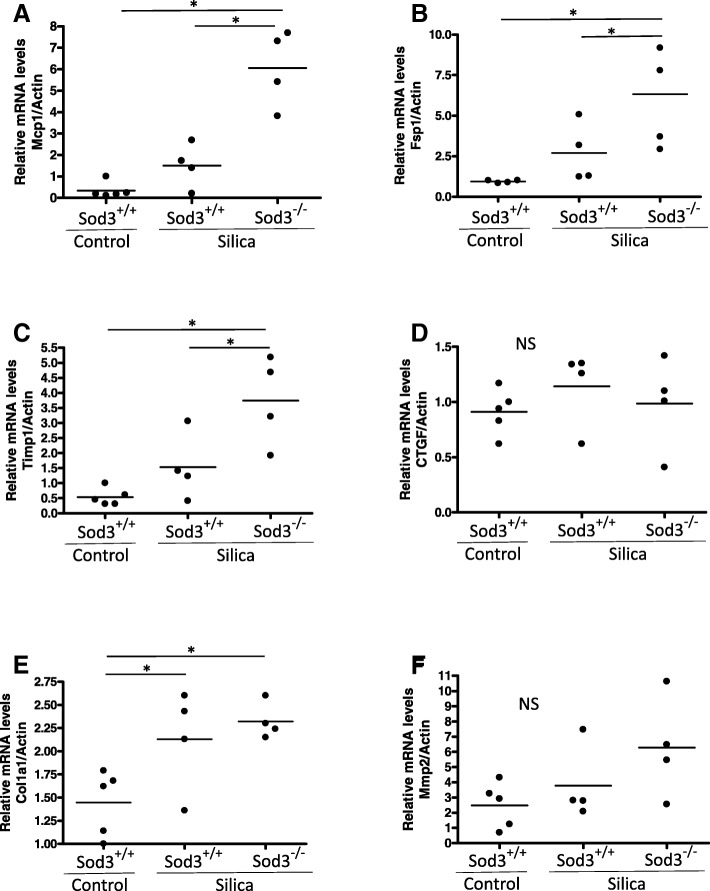
Expression of pro-fibrotic and inflammatory genes in response to silica. Analysis of gene expression in the lung of silica treated mice. Whole lung mRNA levels for (**a**) monocyte chemoattractant protein-1 (Mcp1), (**b**) fibroblast-specific protein 1 (Fsp1), (**c**) tissue inhibitor of metalloproteinases 1 (Timp1), (**d**) connective tissue growth factor (Ctgf), (**e**) collagen type I, alpha 1 (Col1a1), and (**f**) matrix metalloproteinase 2 (Mmp-2) were determined using real-time PCR and normalized to beta-acting expression (β-Actin), *p < 0.05 when compared to control lung, One Way ANOVA with Holm-Sidak post test

The development of lung lesions after exposure of lung to crystalline silica also resulted in alterations in the expression of a number of inflammatory and pro-fibrotic genes. For example, the mRNA expression of collagen type I (Col1a1) was markedly elevated in the right lobe of silica-treated lungs, coinciding with the increased levels of soluble collagen detected using Sircol Collagen Assay. However, no differences between silica-treated Sod3^+/+^ and Sod3^−/−^ mice were observed (Fig. 3e). On the other hand, the mRNA levels of pro-inflammatory cytokine Mcp1, pro-fibrotic fibroblast-specific Fsp1 gene and tissue-remodeling Timp1 gene were significantly upregulated in the lung of silica treated Sod3^−/−^ mice, but not in Sod3^+/+^ mice when compared to control (Fig. 3a-c). The differences in the upregulation of gene expression between Sod3^+/+^ and Sod3^−/−^ were observed only for Mcp1, Fsp1 and Timp1 genes (Fig. 3a-c). Surprisingly, the mRNA levels for growth factor involved in fibrotic transformation of lung (CTGF) and major extracellular matrix remodeling enzyme (Mmp2) did not change after exposure to silica in both mouse strains (Fig. 3d, f). These data suggest that loss of SOD3 expression may predispose the lung to greater inflammatory responses and enhanced pro-fibrotic signaling via distinct pathways.

### Silica exposure is associated with increased neutrophil infiltration in lung, especially around pulmonary arteries

**Fig. 4:**
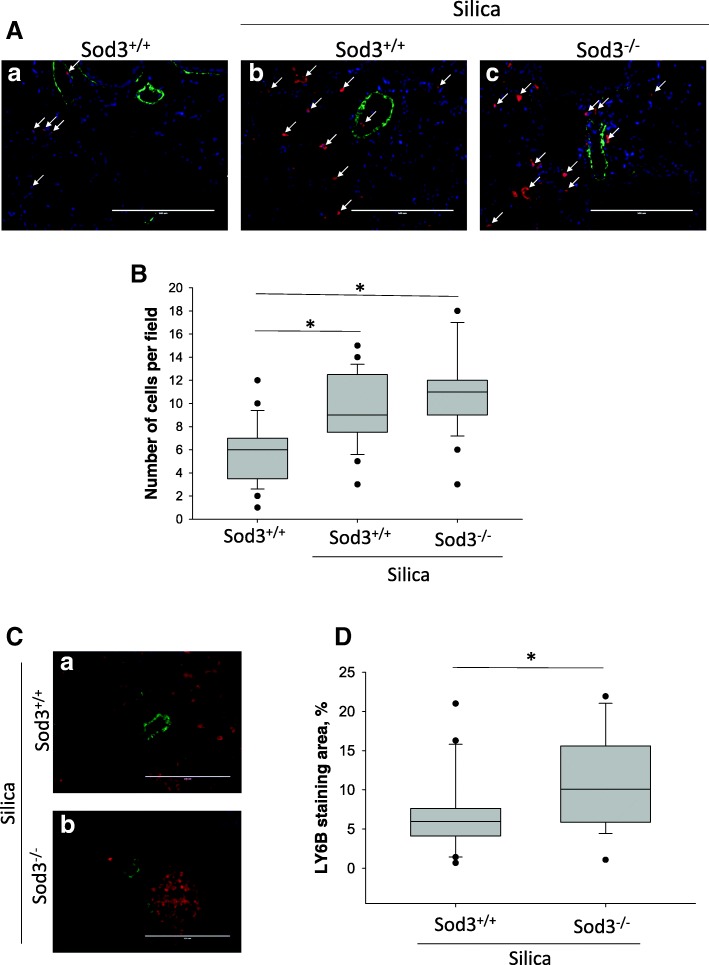
Infiltration of inflammatory cells in the lung of Sod3^+/+^ and Sod3^−/−^ mice treated with silica. **a** Lung sections (5 μm thick) were stained with antibodies specific for macrophage- and neutrophil-specific marker LY-6B (red) and α-smooth muscle actin (green). Nuclei were stained using DAPI (blue). Images were taken using 20x objective. Representative images from unaffected areas of mouse lung from control (image a) and 0.4 mg/kg silica treated (images b and c) mice. Increased infiltration of inflammatory cells into lung found in silica treated lungs of both Sod3^+/+^ and Sod3^−/−^ mice (**panel b**). Bar = 200 μm. *p < 0.05 when compared to control lung, One Way ANOVA with Holm-Sidak post test. **c** Representative images of LY6B positive cells located in close proximity to pulmonary vessels in silicotic nodules. **d** Quantitative analysis of area in close proximity to pulmonary vessels (green) stained with LY-6B (red) specific antibodies. *p < 0.05, *t*-test

To further assess the inflammatory response in the lungs of animals, a marker of neutrophils (LY6B) was examined histologically at 4 weeks (Fig. 4a). In addition to neutrophils, Ly6B marker is expressed on the surface of macrophages polarized toward a proinflammatory phenotype [27]. Silica exposure significantly altered the recruitment of activated macrophages and neutrophils into lung areas that were free of silicotic nodules from 5.8 ± 2.6 cells per field in control mice to 9.5 ± 3.1 cells per field in silica treated mice (*p* < 0.05). However, no significant differences in inflammatory cells recruitment were observed between silica-exposed Sod3^+/+^ and Sod3^−/−^ mice (9.5 ± 3.1 vs 10.8 ± 3.4 cells per field). Since large clusters of LY6B positive cells were detected around pulmonary arteries, we analyzed the areas of LY6B-stained lungs in close proximity to the pulmonary vasculature. Crystalline silica inhalation caused more inflammatory cell accumulation around pulmonary vasculature in SOD3 deficient mice (6.88 ± 4.86%) compared to wild-type littermate (10.74 ± 5.94%) (Fig. 4b). Thus, while silica exposure caused the infiltration of inflammatory cells in the lung, Sod3^−/−^ mice showed enhanced response specifically in the areas that are in close proximity to the pulmonary arteries. This prompted an evaluation of the pulmonary arteries.

### Silica exposure is also associated with pulmonary vascular remodeling and pulmonary hypertension

**Fig. 5:**
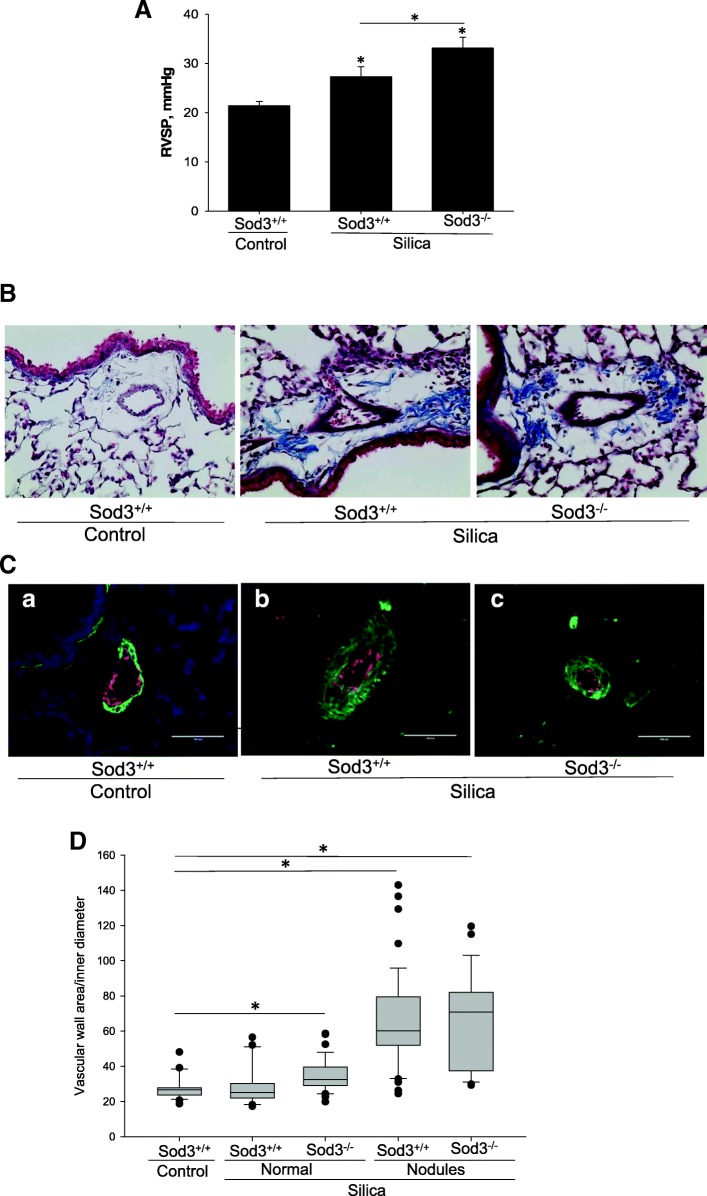
Vascular remodeling in silica treated lungs. **a** Right Ventricular Systolic Pressure (RVSP) was measured using pressure catheter inserted into right ventricle through jugular vein. The results are shown as mean ± SEM, *p < 0.05. **b** Lung sections were stained with Mason’s trichrome to visualize collagen and imaged at 400x magnification. Representative images of pulmonary arteries from control (a) and silica treated Sod3^+/+^ (image b) or Sod3^−/−^ (image c) mice. **c** Lung sections (5 μm thick) were stained with antibodies specific for endothelium-specific von Willebrand Factor vWF (red) and α-smooth muscle actin (green). Nuclei were stained using DAPI (blue). Images were taken using 40x objective. Panel a: representative images of pulmonary arteries from control mice. Panel b and c: images of pulmonary arteries with proliferated smooth muscle cell layer. **d** Quantitative analysis of vascular wall remodeling expressed as ratio of smooth muscle vascular wall area/inner diameter. Bar = 100 μm. *p < 0.05 when compared to control lung, One Way ANOVA with Holm-Sidak post test

Silica-related chronic lung disease has been linked to pulmonary hypertension [28]. The pulmonary hypertension and the above-described increase in perivascular infiltration of activated macrophages and neutrophils detected in the lungs of Sod3^−/−^ animals, justified a more in-depth look at the vascular structures. First, we analyzed the effects of silica on pulmonary blood pressure by measuring right ventricular systolic pressure (RVSP) (Fig. 5a). We found that administration of crystalline silica increased RVSP in wild-type mice from 21.4 ± 0.86 mmHg in control to 27.29 ± 2.01 mmHg in silica treated animals. Sod3^−/−^ mice showed significantly higher RVSP (33.13 ± 2.17 mmHg) after silica treatment compared to Sod3^+/+^ mice (*p* < 0.05) suggesting that SOD3 is protective in the setting of pulmonary hypertension secondary to silicosis.

The magnitude of structural changes in pulmonary vessels, characterized by thickening of the smooth muscle layer, is a good measure of abnormal vascular changes. Therefore, we analyzed pulmonary artery remodeling in Mason’s trichrome stained lung section following silica exposure (Fig. 5b). In addition, we stained pulmonary arteries for alpha smooth muscle actin to measure the hypertrophy of the smooth muscle layer and expressed vascular remodeling as a ratio between smooth muscle surface area divided by inner dimeter (Fig. 5c, d). Pulmonary artery remodeling was measured in the areas not affected by fibrosis and in the silicotic nodules. Interestingly, both strains of mice showed a similar increase in thickness of the vascular smooth muscle layer in structures within the silicotic nodules from 28.67 ± 6.43 in control mice to 65.63 ± 26.55 (*p* < 0.001) in Sod3^+/+^ and to 64.84 ± 26.44 (*p* < 0.001) in Sod3^−/−^ mice after silica exposure (Fig. 5d). However, the measurement of wall thickness in pulmonary arteries located in unaffected areas of the lung indicated remodeling of vascular walls only in Sod3^−/−^ mice, but not in Sod3^+/+^ mice (Fig. 5d). Thus, both hemodynamic and morphological features indicate an important role for SOD3 in development of silica-induced pulmonary hypertension.

### Sod3 deficiency did not change the expression of vascular-specific genes in silicotic lungs

**Fig. 6:**
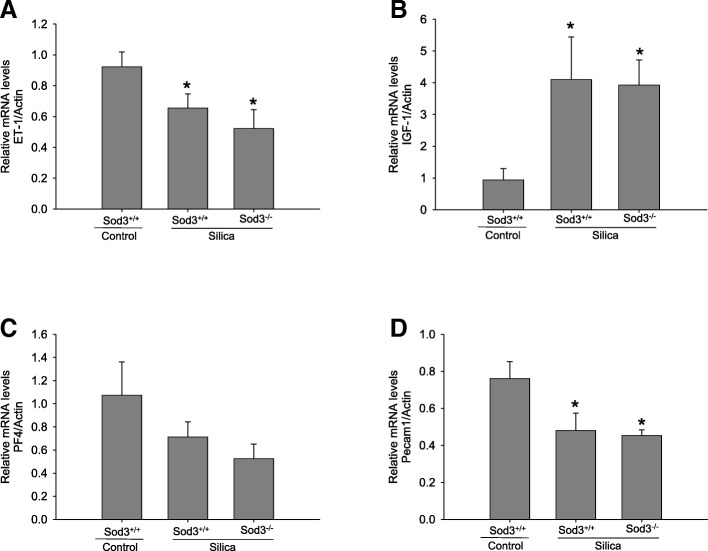
Expression of vascular-specific genes. Analysis of vascular-specific gene expression in the lung of wild-type (Sod3^+/+^) and Sod3 deficient (Sod3^−/−^) mice. Expression of genes was analyzed 28 days following silica instillation. Whole lung mRNA levels for (**a**) endothelin 1 (ET-1), (**b**) insulin growth factor 1 (IGF-1), (**c**) platelet factor 4 (PF4), (**d**) platelet/endothelial cell adhesion molecule 1 (Pecam1) were determined using real-time PCR and normalized to beta-acting expression (Actin). The results are shown as mean ± SEM, *p < 0.05 when compared to control lung, One Way ANOVA with Holm-Sidak post test

Treatment of Sod3^+/+^ and Sod3^−/−^ mice with crystalline silica for 4 weeks attenuated the expression of several endothelium- or smooth muscle-specific genes (Fig. 6). The mRNA levels for ET-1 and Pecam1 were significantly reduced in both strains of mice (Fig. 6a, d) while differences in the expression of PF4 gene between control and silica-treated mice were non-significant (Fig. 6c). Interestingly, the expression of IGF-1 was upregulated 4-fold in silica treated lungs, but no differences were detected between Sod3^+/+^ and Sod3^−/−^ mice (Fig. 6b). These data indicate that crystalline silica might affect the pulmonary vasculature through differential regulation of vascular- specific genes, however the lack of Sod3 in murine lungs did not change the expression of these genes in silica-injured lungs.

## Discussion

Although the protective role of SOD3 in animal models of lung fibrosis has been reported previously [29, 30], its contribution to the development of pulmonary vascular remodeling associated with chronic lung diseases is not well understood. In this study, we show that SOD3 plays an important role in silica-induced lung inflammation and fibrosis. SOD3 also appears important for the development of vascular remodeling and pulmonary hypertension in this model. This work was prompted by the observation that SOD3 expression is reduced in animals exposed to crystalline silica, thereby suggesting a protective role for SOD3. Further work in SOD3-deficient animals revealed increased weight loss, lung structural changes with more pulmonary nodules, enhanced collagen deposition, and alterations in proinflammatory and profibrotic genes when compared to silica-treated wild-type animals. These changes were associated with increased inflammatory cells infiltration in the lungs of Sod3^−/−^ exposed to silica. Interestingly, there was also an increase in infiltrate of activated macrophages and neutrophils in perivascular structures not noted in wild-type animals. This pointed to a role for SOD3 in vascular remodeling for which further studies were performed revealing increased RVSP in all mice exposed to crystalline silica, but an even further increase in RSVP in Sod3^−/−^ animals, which was associated with increased vascular wall thickening. This is important since no previous studies indicated a role for SOD3 in silica-induced pulmonary hypertension. Interestingly, SOD3-deficient mice develop elevated pulmonary blood pressures and profound vascular wall remodeling when compared to neonatal wild-type littermates in the bleomycin model of pulmonary fibrosis [31]. However, the etiology and pathological manifestations of silica-induced changes in lung interstitium and the pulmonary vasculature are substantially different from those induced by bleomycin, thereby requiring further investigation [25]. In addition to the observed increase in RVSP in Sod3^−/−^ mice, we also found differences in inflammatory cell activation and in the rate of hypertrophy of smooth muscle layer in pulmonary arteries.

Our data indicated an excessive deposition of collagen in the silicotic lungs of both strains of mice, although Sod3^−/−^ mice were more susceptible to fibrosis (Fig. 2). This observation was supported by elevated expression of the collagen 1A1 and the fibroblast-specific protein 1 genes (Fig. 3). Fsp1 is related to the family of cytoplasmic calcium-binding proteins and is mostly produced by activated fibroblasts in tissues that undergo excessive remodeling [32]. The Fsp1-positive fibroblasts derived from the lung of mice treated with bleomycin are also characterized by excessive production of alpha1 procollagen. Moreover, under certain inflammatory conditions, Fsp1 is expressed by activated macrophages and other immune cells [33]. Thus, increased levels of Fsp1 in the silicotic lungs may be related to the influx and activation of fibroblasts and immune cells. Our observation of increased collagen deposition coinciding with elevated expression of fibroblast-specific genes is in good agreement with previously published literature.

Moreover, we found increased expression of TIMP-1 gene in Sod3^−/−^ mice exposed to silica. The pattern of TIMP-1 gene expression was similar to MMP-2 gene expression among experimental groups, although the differences in MMP-2 expression did not reached the statistical significance. Similar small increase in Mmp-2 and 8-fold increase of TIMP-1 expression in the lung of bleomycin treated C57BL6J mice has been described previously [34]. It is possible that increase of TIMP-1 expression in Sod3^−/−^ mice can be explained by increased oxidative stress in the lung of these animals [35]. In the lung, TIMP-1 produced mostly by macrophages and epithelial cells. The matrix metalloproteinases and their specific inhibitors are important elements of the tissue remodeling process. Increase in protease inhibitors would promote abnormal tissue repairing pathway resulting in scarring of pulmonary tissue and leading to pulmonary fibrosis [36]. Conversely, the pathological overproduction of MMPs would lead to tissue destruction as observed in emphysema and chronic obstructive pulmonary disease [37]. The role of TIMP-1 in development of pulmonary hypertension and vascular remodeling is controversial. Increased MMP-2 and TIMP-1 expression were identified in pulmonary artery smooth muscle cells isolated from idiopathic PAH patients [38]. Vieillard-Baron et al. reported that inhibition of MMPs activities by intratracheal delivery of TIMP-1 exacerbated pulmonary hypertension and increased muscularization and periadvetitial collagen accumulation in distal arteries of rats subjected to chronic hypoxia [39]. Other studies found evidence that ECM degradation may be essential to smooth muscle cells migration and proliferation [40]. Moreover, targeted delivery of TIMP in luminal cells inhibited neointimal formation in human saphenous veins [41]. While the reason for the discrepancy between these studies it not clear, it may be ascribable to differences in studied vascular structures. Since we investigated vascular remodeling in fibrotic lungs, it is also possible that increased hypertrophy of smooth muscle layer in pulmonary arteries can be stimulated by rise in pulmonary artery wall tension due to excessive deposition of extracellular matrix [42].

We also found increased influx of inflammatory cells in the lungs of mice challenged with silica. We used Ly6B antigen as a neutrophil marker, however, recent data suggested that Ly6B could also be expressed by specific Ly6B^high^ subset of proinflammatory macrophages. This subtype of activated macrophages exacerbate fibrotic renal injury by secreting extracellular matrix compounds including fibronectin and collagen [27]. They also produce proinflammatory cytokines and chemokines that help to recruit and differentiate additional macrophages into sites of injury creating perpetual injurious cycle. Interestingly, the number of Ly6B positive inflammatory cells in the non-affected areas of silica-exposed lungs was significantly higher in Sod3^−/−^ mice suggesting an important role of SOD3 in chemotaxis of these cells into the pulmonary intersitium. Previous studies supported a role for superoxide in inflammatory cell migration [43] and a beneficial anti-inflammatory role for SOD3 in this process [44]. Moreover, acute deletion of SOD3 alone leads to massive infiltration of granulocytes, T cells, and natural killer cells in lung [45]. Crystalline silica produces its damaging effects through activation of immune cells and production of reactive oxygen species (ROS) burst [46, 47]. In turn, elevated ROS levels can lead to epithelial and vascular cell damage promoting fibrogenic remodeling of lung interstitium and proliferation of cells in the vascular media. SOD3 is a potent anti-inflammatory agent and an inhibitor of inflammatory cell influx in response to asbestos, bleomycin, hyperoxia and lipopolysaccharides [48–51]. Therefore, loss of SOD3 from the lung of genetically-modified animals may predispose pulmonary epithelial and vascular cells to ROS-induced damage due to diminishing antioxidative protection. On the other hand, infiltration of macrophages and neutrophils specifically around remodeled pulmonary vessels may deliver a new load of SOD3, thereby alleviating ROS-induced damage, while normalizing signaling pathways responsible for vascular cell proliferation and hypertrophy Interestingly, the increased influx of immune cells in Sod3^−/−^ mice coincided with an elevated expression of Mcp1 gene, which is one of the key chemokines that regulate migration and infiltration of monocytes and macrophages into the lung [52]. The lack of experimental evidences supporting the role of ROS in observed exacerbation of pulmonary fibrosis and pulmonary hypertension in Sod3^−/−^ mice is a major limitation of this study.

The effects of crystalline silica and other environmental and occupational particulate matter on the expression of antioxidant enzymes in the lung are not well known. We observed a decrease in the expression of SOD3 in the lungs of silica-treated mice at day 28. This attenuated expression of SOD3 is in good agreement with reduced expression of SOD3 in the lung intersitium of wild-type mice treated with bleomycin and asbestos [48, 53] and with decreased SOD3-specific staining in IPF patients [54]. On the other hand, others reported differential regulation of superoxide dismutases in the lung of silica-treated rats [55]. Kim et al. indicated that SOD3 gene expression in the lung was elevated compared to control animals starting from day 3 and up to 90 days following silica administration. This observation contrasts with ours when analyzing SOD3 expression 28 days following silica exposure in mice. There are several possible explanations for this discrepancy. First, different species of rodents (mice vs rats) were used in these studies. Second, the dosage of administered silica and differences in chemical and morphological characteristics of crystalline silica may lead to different responses. For example, it has been shown that crystalline silica with a geometric mean diameter of 1.8 μm produces more profound effects on inflammatory cell infiltration into the bronchoalveolar lavage fluid harvested from lungs when compared to silica with a diameter of 0.7 μm [56]. Further studies will be required to evaluate these differential findings and how they are relevant to the human condition.

A key finding of this work is that the fibrotic changes observed in our model were accompanied by significant vascular changes. The analysis of vascular remodeling in silica-treated animals revealed increased thickening of smooth muscle layer in the pulmonary arteries of silicotic lungs. Interestingly, no differences in smooth muscle hypertrophy were detected in the fibrotic areas between Sod3^+/+^ and Sod3^−/−^ mice, thereby suggesting a differential role for SOD3 in vascular and non-vascular structures. The profound remodeling of the vascular media in fibrotic areas of lung has been described previously by others and us [25, 31]. It is intriguing to note that the lack of SOD3 in these remodeled pulmonary vessels was not accompanied by changes in smooth muscle proliferation, while enhancing collagen deposition and cells infiltration. On the other hand, Sod3^−/−^ mice developed significant thickening of the pulmonary artery walls located in unaffected areas of the lung when compared to wild-type littermates treated with silica. To our knowledge, this is the first description of differential vascular remodeling in non-fibrotic and fibrosing lung compartments in the setting of silica exposure.

To further investigate the potential molecular mechanisms involved in vascular remodeling, we measured the expression of several vasculature-specific genes. There were no significant differences in expression of ET-1, PECAM-1 and PF4 genes between Sod3^+/+^ and Sod3^−/−^ mice, although silica treatment attenuated their expression in both mouse strains. Therefore, observed exacerbation of vascular remodeling and pulmonary hypertension in Sod3 deficient mice is not associated with differential regulation of these factors after exposure to silica. In addition, we quantified mRNA levels of IGF-1 since this growth factor might be a potent mediator of vascular growth responses in settings of pulmonary hypertension. Indeed, IGF-1 was significantly elevated in silicotic lungs of both mouse strains indicating important role of IGF-1 signaling in pulmonary response to crystalline silica. Similar induction of IGF-1 expression in the lung of neonatal mice exposed to hypoxia has been described previously [57]. Although increased signaling through IGF-1 in response to silica administration is observed in this study, the other pathways triggered by EGF, FGF, and PDGF were not examined and need further investigation.

Our findings may also have a clinical relevance. The pulmonary hypertension in patients with silicosis, COPD, lung fibrosis and sarcoidosis is associated with higher mortality rate [9–11]. The elucidation of mechanisms by which silicosis may lead to elevated pulmonary pressure may provide novel therapeutic strategies. These findings further implicated important role of Sod3 in progression of silica induced lung fibrosis and pulmonary hypertension.

## Conclusions

In conclusion, we report that mice lacking SOD3 developed increased RVSP and pulmonary vascular remodeling associated with enhanced collagen deposition, increased influx of inflammatory cells, and alterations in proinflammatory and profibrotic gene expression. Our results suggest that SOD3 play an important role in silica-induced lung fibrosis and vascular remodeling in the lung and provides new insight into the role of antioxidant defense mechanisms responsible for protecting the lung from the harmful effects of air pollution and occupational hazards.

## Abbreviations

COPD: Chronic obstructive pulmonary disease
CTGF: Connective tissue growth factor
ET-1: Eendothelin 1
FSP-1: Fibroblast-specific protein 1
LY-6B: Lymphocyte antigen 6 complex, locus B
MCP-1: Monocyte chemoattractant protein – 1
MMP-2: Matric metallopeptidase – 2
PH: Pulmonary hypertension
RVSP: Right ventricular systolic pressure
TIMP-1: Tissue inhibitor of metalloproteinase – 1
TNF-α: Tumor necrosis factor alpha
